# Understanding the impact of rapid antigen tests on SARS-CoV-2 transmission in the fifth wave of COVID-19 in Hong Kong in early 2022

**DOI:** 10.1080/22221751.2022.2076616

**Published:** 2022-05-23

**Authors:** Zhanwei Du, Linwei Tian, Dong-Yan Jin

**Affiliations:** a School of Public Health, LKS Faculty of Medicine, The University of Hong Kong, Pokfulam, Hong Kong, People’s Republic of China; b School of Biomedical Sciences, LKS Faculty of Medicine, The University of Hong Kong, Pokfulam, Hong Kong, People’s Republic of China

**Keywords:** COVID-19, SARS-CoV-2, rapid antigen tests, home quarantine, superspreading

## Abstract

The fast-spreading Omicron variant of SARS-CoV-2 overwhelmed Hong Kong, causing the fifth wave of COVID-19. It remains to be determined what mitigation measures might have played a role in reversing the rising trend of confirmed cases in this major outbreak. The government of Hong Kong has launched the mass rapid antigen tests (RAT) in the population and the StayHomeSafe scheme since February 2022. In this study, we examined the impact of the mass RAT on disease transmission and the case fatality ratio. It was suggested that the implementation of RAT plausibly played a role in the steady decrease of the effective reproduction number, leading to diminished SARS-CoV-2 transmission. In addition, we projected the disease burden of the outbreak in a scenario analysis to highlight the necessity of the StayHomeSafe scheme in Hong Kong. The Omicron outbreak experience in Hong Kong may provide actionable insights for navigating the challenges of COVID-19 surges in other regions and countries.

## Introduction

Coronavirus Disease 2019 (COVID-19) continues its global march outward in 2022 and has been a global public health and medical crisis, resulting in 504 million reported cases and 6.2 million deaths as of 18 April 2022 [1]. This is made worse by the emergence of multiple SARS-CoV-2 variants (e.g. Delta and Omicron), which may result in increased transmissibility and lower vaccine effectiveness [2].

In Hong Kong, the first COVID-19 local untraceable Omicron case was confirmed on 7 January 2022 [3]. Then, the fast-spreading BA.2 subvariant of the Omicron variant of SARS-CoV-2 swept Hong Kong and triggered the fifth wave of COVID-19. A total of 1.2 million cases and 9176 deaths were recorded in Hong Kong as of 20 April 2022 [4], although 80% and 41% of the population had been immunized with the 2nd and 3rd vaccine dose, respectively [5].

A series of public health and social measures (PHSMs) have been put in place since the beginning of 2020 in Hong Kong, including the testing and tracking of suspected cases, the isolation of confirmed cases, along with strict travel and border restrictions [4], which were successful in suppressing the past four local outbreaks in Hong Kong, but failed to avert the massive epidemic as the fifth wave started in January 2022 as a result of an accidental infection that occurred in a quarantine hotel [6].

For early identification and isolation of infected individuals, the government of Hong Kong has purchased large quantities of rapid antigen test (RAT) kits for use in various age groups of population [7]. PHSMs including school closures [8], work restrictions [9], and travel bans [4] were reimposed in January or February 2022 to locate and quickly isolate persons who have been exposed to the virus. RAT alone was officially recognized as a diagnostic test for SARS-CoV-2 infection on 26 February 2022 [10].

Although many temporary community isolation and treatment facilities (compartment or makeshift hospitals) supported by Mainland China have been rapidly built since 20 January 2022 to isolate infected individuals [11], the increasingly high number of cases still force a choice between home quarantine and health services disruption. As a viable cost-effective alternative for infected people and their close contacts, the StayHomeSafe scheme was launched on 8 February 2022 for individuals to be quarantined at home for 14 days or 4 days, following an assessment [12].

Under the dynamic zero COVID policy, the central government of China responded to Omicron outbreaks in March 2022 with rapid lockdowns, mass testing, and travel bans whenever clusters had emerged. As of late April 2022, the ongoing fifth wave of COVID-19 in Hong Kong is nearing its end. We suppose that the community-wide low-cost rapid SARS-CoV-2 antigen tests have played a key role in reducing virus transmission in Hong Kong during the fifth wave, coupled with the reduced health burden from the expansion of isolation programmes through StayHomeSafe [13]. In this study, we evaluate the impact of the mass rapid antigen testing, together with PHSMs and vaccination, on disease transmission. We also project the health burden created by the fifth wave to reveal the values of the StayHomeSafe scheme in Hong Kong. The lessons learned from combating the Omicron outbreak in Hong Kong may provide actionable insights for navigating the challenges in other parts of the world including Mainland China.

## Materials and methods

### Epidemic data

Starting from 26 February 2022, those who tested positive for SARS-CoV-2 virus at least once by nucleic acid tests or RAT in Hong Kong are considered confirmed cases [10]. Previously, RAT-positive cases had to submit additional samples for nucleic acid testing to confirm the results. But to avoid resource waste and time delay in finding COVID-19 cases in the fifth wave of Hong Kong, those cases tested positive at least once by RAT, whether distributed by the government or on their own purchase, do not need to seek confirmation by nucleic acid tests from 26 February 2022 [7,10]. We collected the daily numbers of all newly reported COVID-19 cases confirmed by either RAT or nucleic acid tests, cumulative number of individuals fully vaccinated or with boosters, in Hong Kong from 1 January to 26 March 2022 [4] for analysis.

### Government response data

We collected the daily COVID-19 Stringency Index [14], which is a composite measure of nine response metrics (school closures, workplace closures, public event, public gatherings, public transport, stay-at-home requirements, public information campaigns, internal movements, and international travel controls), in Hong Kong from 1 January to 26 March 2022 to represent the control measures in Hong Kong. This index ranges between 0 and 100 [14], with a higher score indicating a more stringent response (i.e. 100 = the most stringent response).

### Epidemic modelling

Following the COVID-19 Covasim model [15], we assigned populations as susceptible (*S*), exposed (*E*, infected but not yet infectious), pre-symptomatic (*P*), asymptomatic (*A*), mild (*I*_1_), severe (*I*_2_), critical cases (*I*_3_), recovered (R), or died (D). A susceptible individual (S) progresses to exposure (E) and then becomes either presymptomatic (P) or asymptomatic infectious (A) with probability *p_sym_* and 1 – *p_sym_*. Those presymptomatic individuals will have mild, and then severe, and critical symptoms with probability *p_sev_* and *p_cri_*, respectively. Most of these symptomatic individuals will recover (R), while a proportion *p_dea_* of *I*_3_ will die (D). Age-stratified parameter definitions are in Table S1, together with default duration parameters (Table S2) in the Covasim model [15]. We initialized each stochastic simulation with 0.1% randomly exposed individuals in each age-stratified population in Hong Kong [16]. The transmission rate of infectious individuals was estimated for each study basic reproduction number (*R_0_*) using the method of the next-generation matrix [17].

## Results

**Figure 1.**
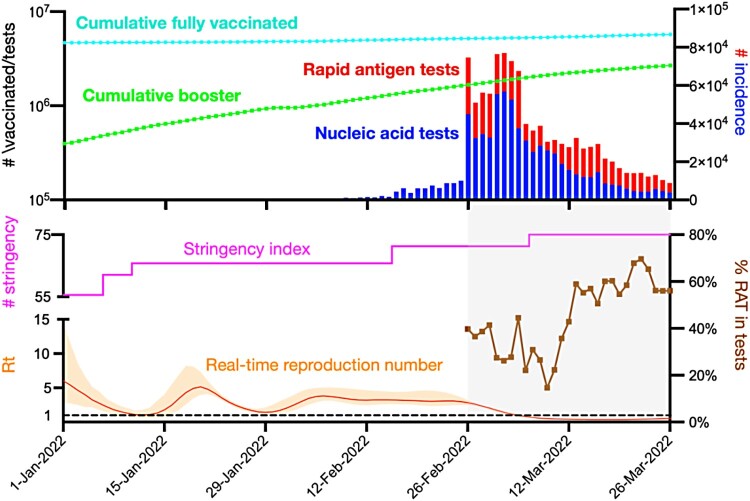
COVID-19 incidence, vaccination, containment, and transmission in Hong Kong from 1 January to 26 March 2022. The dotted black line denotes the threshold of *R_t_* at the value of 1.0. The mean *R_t_* declined to below 1.0 after 5 March 2022. Orange line and shading indicate the mean and 95% confidence interval bounds of the real-time reproduction numbers. The sudden drop of *R_t_* occurred on the day of 26 February, which coincided with the implementation of mass RAT testing, and it decreased continually after the implementation of mass RAT testing.

In the study period of the fifth wave, there were 1,107,821 cases and 6888 deaths, compared to 12,631 cases and 213 deaths in the previous four waves (Figure 1). On 3 March 2022, there were 76,991 newly reported cases, among which 56,827 and 20,164 cases were diagnosed by nucleic acid tests and RAT, respectively. The percentage of confirmed cases by RAT increased from 40% to 70% within 24 days (till 22 March 2022). The stringency index increased from 55 to 75, the highest value in Hong Kong during the battle against COVID-19.

The government of Hong Kong recognized and implemented the mass RAT testing programme on 26 February 2022, along with the existing platform for nucleic acid testing. The percentage of cases confirmed by RAT in all detected cases decreased initially and then increased to 56% after 9 March 2022. The real-time reproduction number (*R_t_*) was 6.03 (95% CI: 2.80, 14.29) at the beginning of the fifth wave, and decreased substantially on the day of 26 February, which coincided with the implementation of mass RAT testing. *R_t_* has dropped to below 1.0 since 5 March 2022.

**Figure 2.**
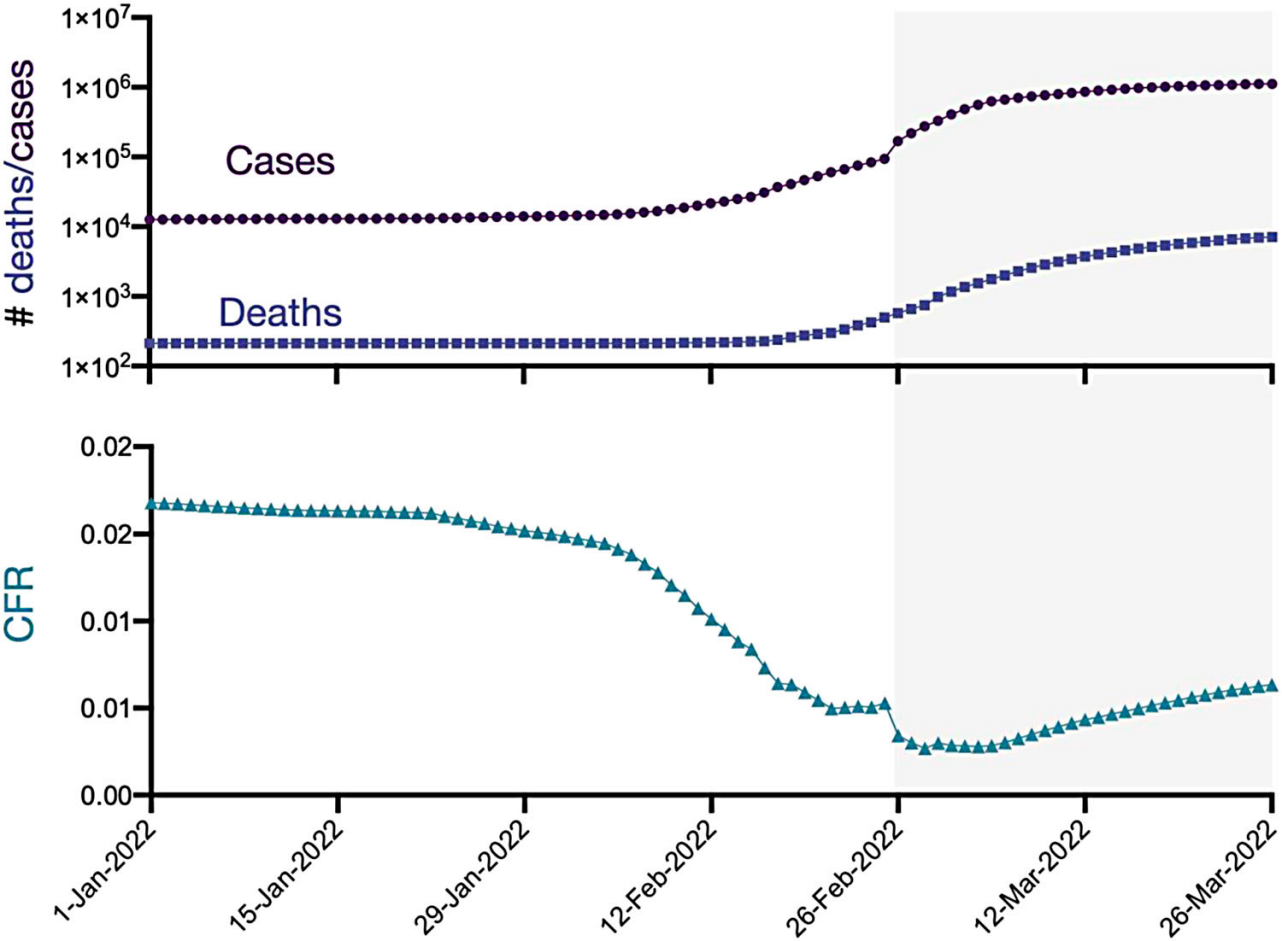
Case fatality ratio (CFR) of the fifth wave of COVID-19 in Hong Kong from 1 January to 26 March 2022. Grey shading denotes the period after the start of mass RAT testing in Hong Kong on 26 February 2022.

**Figure 3.**
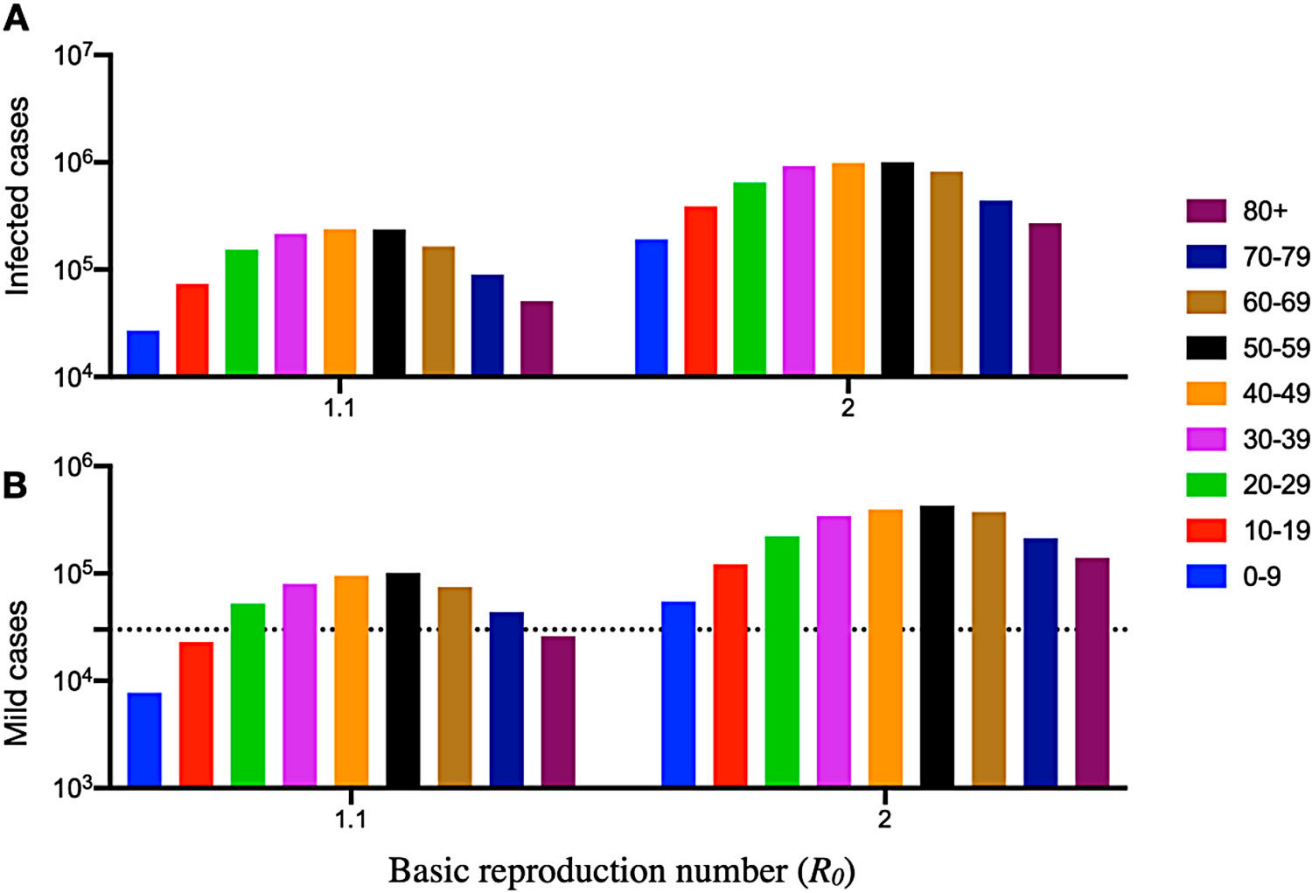
Projected age-stratified disease burden of COVID-19 in Hong Kong. For each basic reproduction number ranging from 1.1 and 2, we estimated the mean in (A) total infections and (B) mild infections using COVID-19 epidemic modelling. The dotted line indicates the number of 30,105 available hospital beds in all public hospitals in Hong Kong as of March 2022 [18].

We also found that ramping-up mass asymptomatic testing for SARS-CoV-2 in Hong Kong was associated with a lower case fatality ratio (CFR) (Figure 2), which declined substantially from 0.005 on 25 February to 0.003 on 26 February 2022, when the programme of RAT testing was initiated in Hong Kong on 26 February 2022. We estimated the age-stratified numbers of infected, severe and mild cases as well as deaths across a range of transmission scenarios for five different effective reproduction numbers (*R_0_*) (1.1, 1.5, 2, 2.5, and 3). Take *R_0_* at 1.1 as an example, we estimated that there would be 0.5 million mild cases and 1.25 million cases in total, in which there could be 10,000 deaths in Hong Kong (Figure 3, Figure S1).

## Discussion

Our analysis of the fifth wave COVID-19 in Hong Kong suggests that the aggressive SARS-CoV-2 RAT testing programme could contribute to the reduction in transmissibility, which is consistent with an earlier study of the USA scenario [19]. High-quality RAT kits could detect infected cases with high accuracy. For example, the BinaxNOW RAT kits, which cost roughly ∼HK$50 per test, provide a sensitivity of 97.1% and specificity of 98.5% [20,21]. In order to reduce the daily backlog of samples awaiting RT–PCR confirmation by health officials, the Hong Kong government has begun relying on RAT kits to diagnose SARS-CoV-2 infection and sped up the process of confirming and isolating infected people in the fifth wave. RAT testing can now be finished at home within 20 min, rather than at a community testing centre or by utilizing a specimen bottle to collect saliva samples and waiting for days to receive the result of a nucleic acid test. As the cost of testing for SARS-CoV-2 is rapidly decreasing in Hong Kong from ∼HK$100 in February 2022 to ∼HK$10 in April 2022, RAT testing has become an increasingly cost-effective and impactful solution to mitigate the enormous threat of the COVID-19 pandemic, especially with sufficient supply of RAT kits in the Hong Kong market ascribed to strong support from the central government of China [7].

With the implementation of the mass RAT testing programme on 26 February 2022, the total number of cases confirmed by either RAT or nucleic acid tests increased suddenly, nearly 7-fold of that on 25 February 2022. Hong Kong's ability to screen out the infected cases as well as to isolate and treat the infected people has been greatly enhanced by the strong support from the central government of China since January 2022 [11], which was probably one of the reasons underlying this sudden change in numbers of confirmed cases. Mechanistically, mass RAT testing can complement the nucleic acid tests to identify and isolate asymptomatic and symptomatic cases and cut their potential transmission chains. In our prior studies in USA [19,22], we have found the high epidemiological impact of mass RAT testing on the reduction of cases, hospitalizations, and deaths, especially when the frequency of RAT testing is high (e.g. daily) under a low transmission scenario (e.g. the initial effective reproduction number is 1.2). However, many public health and social measures have been put in place in different periods of the fifth wave. Although we find the coincidence of the sudden drop in real-time reproduction number with the implementation of mass RAT testing (Figure 1), we have to caution that other measures enforced around the same time and heightened vigilance from the general public in view of the peak number of confirmed cases probably also contributed to the reduced transmissibility. However, the social distancing measures, the stringency index, and the population screening programmes other than the mass RAT testing did not have a sudden change around 26 February 2022.

**Figure 4.**
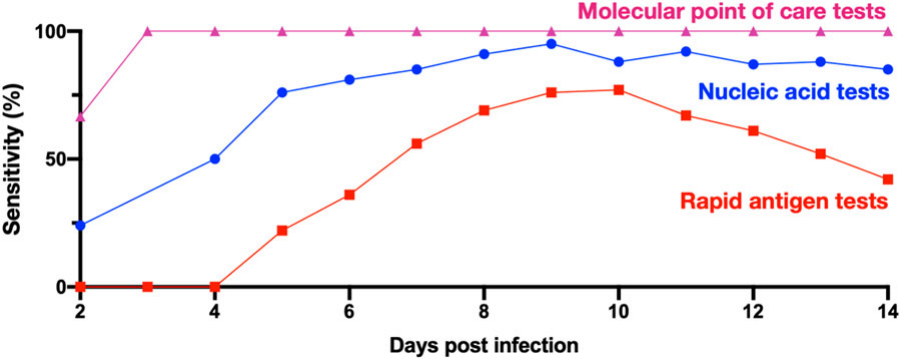
Sensitivity of nucleic acid tests, rapid antigen tests, and molecular point-of-care (POC) tests. The red, blue, and pink lines denote the sensitivity over days post infection of nucleic acid tests [23] and rapid antigen tests [23], molecular point of care tests, respectively [24–26]. The sensitivity denotes the probability of having a positive test result given SARS-CoV-2 infection. Molecular techniques, such as RT–PCR, are considered the gold standard to identify SARS-CoV-2 but need days to generate results [24–26]. The POC testing has the potential to enhance this approach by generating high-sensitivity results within 20 minutes [24–26]. Molecular POC tests can be rapid nucleic acid tests, antigen tests and antibody tests performed or interpreted in various POC settings by healthcare professionals other than the person being tested.

During the fifth wave, there was discussion on whether or not universal RNA testing should be mandated. This is extremely costly and may result in superspreading events when people gather by lining up for testing. However, when the mandatory universal testing is scheduled, the combination of RT–PCR, RAT tests, and molecular point-of-care (POC) tests for the same individual during the same period of time may be a viable option. We should realize that the sensitivity of RAT kits, e.g. QuickVue At-Home OTC COVID-19 Test; Quidel Corporation, increases from 20% post the fifth day to 80% post the eighth day after infection [23]; the high-quality RT–PCR test reaches 80% post the fourth day after infection [23], while the molecular POC test has a high sensitivity with close to 100% post the third day after infection [24,25]. Testing an infected individual with two or three kinds of tests together at the same period of time could result in a high sensitivity close to 80% at least after the fourth day post infection (Figure 4).

Most if not all RAT-positive cases in Hong Kong have been subjected immediately to quarantine either at home or at public quarantine facilities according to the need and intention of the person. In the study period of the fifth wave, 1.1 million cases would need quarantine facilities, but there were a total of 30,105 hospital beds in all public hospitals in Hong Kong as of March 2022 [18]. Isolation and quarantine at home was a viable choice for Hong Kong in the fifth wave. Without the StayHomeSafe scheme launched on 8 February 2022, we estimated that there would be 0.5 million mild cases at an *R_0_* of 1.1, which would overwhelm the Hong Kong public health system easily (Figure 3).

RAT-positive people who are likely to develop into severe cases received pharmaceutical interventions immediately in Hong Kong. Although the Hospital Authority of Hong Kong has introduced two oral drugs, Paxlovid and Molnupiravir, they have been prescribed mainly to ease symptoms and reduce hospitalizations of patients within five days of symptom onset. While these pharmaceutical interventions may reduce the infectivity of infected cases [27], vaccination programme remains the best means to reduce infection susceptibility and infectivity.

As of 17 April 2022, only 63.93% of the elderly aged 80 or above in Hong Kong received at least the first dose of SARS-CoV-2 vaccine, which is similar to the proportion of children aged 0–19 (62.06%) [28]. Even in the case of adequate vaccine supply in Hong Kong, some of the elderly still have concerns over vaccine safety, leading to low vaccination rates [5]. In the potential sixth wave due to an emerging variant, a large number of vaccines are needed in a short period to activate/reactivate the immunity of all populations against the new variant, especially for children and elders. The experts of the Hong Kong government have recommended reduced doses of BioNTech vaccine for children aged 5- to 11-years-old against the local transmission of the Omicron variant, which has been approved in the US and the UK [29,30]. This option should also be seriously considered in Mainland China upon the approval of BioNTech vaccine.

The immediate isolation of RAT-positive individuals is also critical to the prevention of superspreading, which is one driver of COVID-19 outbreaks. With high potential of superspreading, larger outbreaks could still occur even when an epidemic seems to be under control. Early epidemics of COVID-19 are likely fuelled by superspreading events, which can be mitigated by PHSMs. In February 2020, Japan acknowledged the importance of superspreading, established cluster-focused backwards contact tracing, and promoted awareness of persons at risk of infection by restricting higher risk locations: these practices were reflected in the guidelines by the World Health Organisation’s Western Pacific Region in July 2020 [31].

For SARS-CoV, SARS-CoV-2 and MERS-CoV, a small number of persons are responsible for the majority of infections. One hospitalized patient in Beijing, China, who was infected by SARS-CoV in 2003, spread the virus to 76 other people [32]. In South Korea, 154 people were infected with MERS-CoV by five patients in 2015 [33]. In Hong Kong, 19% and 20% of cases were expected to seed 80% of all local transmission in both early 2020 [34] and the fifth wave [35], with 8.9% of cases accounting for 80% of infections in China [36]. According to the recent systematic review [37], many studies indicate that Mainland China has a higher potential of superspreading than Hong Kong. The potential of superspreading would decrease with the decreasing reproduction number [37]. The government may need to accurately estimate the superspreading potential for determining the potential need for, and intensity of, PHSMs needed for disease control. Relaxing PHSMs to reopen societies becomes conceivable when superspreading potential is minimal [38].

We typically use the statistical metric of dispersion parameter, to characterize the potential for superspreading in transmissibility [39], with small values denoting a high potential of superspreading. The dispersion parameter would decrease slightly with the increasing reproduction number [37], denoting the higher potential of superspreading. For COVID-19, the dispersion parameter was 0.55 (95% CI: 0.30, 0.79) globally as per the recent systematic review [37]. In Hong Kong, it was 0.43 (95% CI: 0.38, 0.49) and 0.33 (95% CI: 0.17–0.62) during January to April 2020 [34] and in the fifth wave [35], respectively. In mainland China, it was reported to be 0.58 (95% CI: 0.35,1.18) during January and February 2020 [36].

The successful use of RAT testing in the fifth wave of COVID-19 in Hong Kong is highly relevant to strategic planning of the combination of diagnostic tests in the fight against the Omicron variant in Chinese cities including Jilin and Shanghai. In Jilin, the latest COVID-19 Omicron outbreak has had a devastating impact. To stop the spread of COVID-19, China announced a travel quarantine for the province of Jilin on 14 March 2022. This was the first province-wide travel restriction imposed by China since the lockdown of Wuhan and Hubei at the start of the 2020 pandemic. As of late April 2022, the Omicron outbreak in Hong Kong faded out while the Omicron variant continues to cause the most severe COVID-19 outbreaks in China since the first epidemic wave from Wuhan, resulting in lockdowns in the cities, including Shanghai, Shenzhen, and Jilin. Although the Hong Kong experience to combat the Omicron outbreak could be exemplary to Mainland China, the mass RAT testing requires huge amounts of low-cost rapid SARS-CoV-2 antigen test kits and multifaceted distribution schemes. Daily testing across Mainland China would require over 1.4 billion tests per day, while there might be less than 0.1 billion RAT kits produced per day currently. The RAT testing frequency could be customized for a particular region, depending on the local transmission dynamics and immunity levels [22].

The apparently higher CFR in Hong Kong than that in Mainland China might partially be attributed to the different definitions of COVID-19 and COVID-19 death, along with the high proportion of unvaccinated elderly in Hong Kong [13]. People who die within 28 days of the first positive specimen collection day with SARS-CoV-2, but may be unrelated to COVID-19, were also counted as COVID-19 death cases in Hong Kong [40]. In contrast, the compulsory testing in Mainland China helps to detect a large quantity of asymptomatic individuals, which would also contribute to a relatively lower estimate of CFR.

Our study has some limitations. While we are certain about the qualitative findings, the quantitative results are based on a number of simplifying assumptions. Our data did not include undetected cases, especially those mild cases before mass RAT testing; nor did we consider the impact of vaccination in epidemic modelling explicitly, which was reflected by the low level of initial basic reproduction numbers potentially. In addition, we did not consider the indirect impact of public fatigue in Hong Kong due to the long period of the fight against COVID-19 [41,42].

Our analysis provides another perspective to understand the factors underlying the decrease of effective reproduction number of SARS-CoV-2 transmission in the fifth wave of COVID-19 in Hong Kong. Our findings suggest an important role of RAT testing in the control of the outbreak. Without a city lockdown or the universal mandatory viral RNA testing, the fifth wave of COVID-19 subsided in Hong Kong. In addition, the quarantine at public facilities was not compulsory but based on the need and intention of the infected subjects. Actually, more than 700,000 infected cases were quarantined at home and contact tracing was also suspended when the rising trend of confirmed cases was reversed.

## Conclusions

In the fifth wave of COVID-19 in Hong Kong, the widely administered RAT kits probably played a major role in detecting asymptomatic individuals, along with the RT–PCR tests, resulting in a lower CFR and a diminishing real-time reproduction number. In addition, the StayHomeSafe programme played an important role in easing the isolation and treatment burden of hospitals in Hong Kong.

## Supplementary Material

Supplemental Material
