# Supplementary material for: Understanding the impact of rapid antigen tests on SARS-CoV-2 transmission in the fifth wave of COVID-19 in Hong Kong in early 2022
Source: Emerg Microbes Infect. 2022 May 23;11(1):1394–401. doi: 10.1080/22221751.2022.2076616 (PMC9132401; doi:10.1080/22221751.2022.2076616)
Supplement: Supplemental Material [file TEMI_A_2076616_SM8827.docx]

**Supplementary materials**

**Table S1**. Age-stratified epidemiological parameters in our epidemic model

| Age groups | [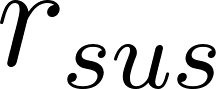](https://www.codecogs.com/eqnedit.php?latex=r_%7Bsus%7D#0): relative susceptibility to infection | [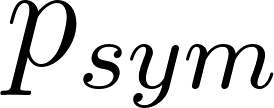](https://www.codecogs.com/eqnedit.php?latex=p_%7Bsym%7D#0): probability of developing symptoms * | [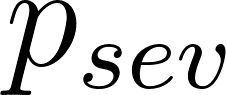](https://www.codecogs.com/eqnedit.php?latex=p_%7Bsev%7D#0): probability of developing severe symptoms | [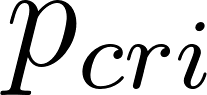](https://www.codecogs.com/eqnedit.php?latex=p_%7Bcri%7D#0): probability of developing into a critical case ^+^ | [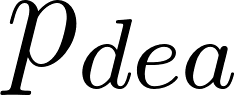](https://www.codecogs.com/eqnedit.php?latex=p_%7Bdea%7D#0): probability of death ^+^ |
| --- | --- | --- | --- | --- | --- |
| 0-9 | 0.3400 | 0.2857 | 0.0005 | 0.7746 | 0.5164 |
| 10-19 | 0.6700 | 0.3143 | 0.0017 | 0.6639 | 0.1660 |
| 20-29 | 1.0000 | 0.3429 | 0.0072 | 0.2887 | 0.0802 |
| 30-39 | 1.0000 | 0.3714 | 0.0208 | 0.1550 | 0.0477 |
| 40-49 | 1.0000 | 0.4000 | 0.0343 | 0.1660 | 0.0753 |
| 50-59 | 1.0000 | 0.4286 | 0.0765 | 0.2714 | 0.0771 |
| 60-69 | 1.0000 | 0.4571 | 0.1328 | 0.4334 | 0.0912 |
| 70-79 | 1.2400 | 0.4857 | 0.2066 | 0.6056 | 0.1655 |
| 80+ | 1.4700 | 0.5143 | 0.2457 | 0.8951 | 0.5224 |

* The probability of developing symptomatic diseases for vaccinated individuals is 0.4, estimated by the Omicron outbreak in Kwai Chung Estate in Hong Kong [[43]](https://paperpile.com/c/6RLUaH/0Vx56). Given that over 80% individuals in Hong Kong had been vaccinated by February 2022 [[40]](https://paperpile.com/c/6RLUaH/WyHFc), we scale [
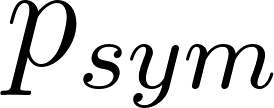
](https://www.codecogs.com/eqnedit.php?latex=p_%7Bsym%7D#0) with values in the Covasim model [[15]](https://paperpile.com/c/6RLUaH/B0W9U) to match its mean value over age groups to 0.4.

+ We scale [
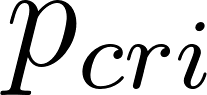
](https://www.codecogs.com/eqnedit.php?latex=p_%7Bcri%7D#0) and [
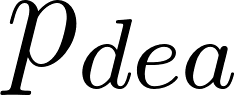
](https://www.codecogs.com/eqnedit.php?latex=p_%7Bdea%7D#0) with values in the Covasim model [[15]](https://paperpile.com/c/6RLUaH/B0W9U) to match Case fatality rate (CFR) by age group estimated by the Centre for Health Protection of the Department of Health in Hong Kong on 16 April 2022 [[4](https://paperpile.com/c/6RLUaH/FIwEu)].

**Table S2**. Time period parameter for state transitions in our epidemic model

| Parameter | Description | Value (day) |
| --- | --- | --- |
| [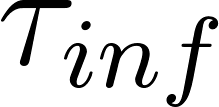](https://latex-staging.easygenerator.com/eqneditor/editor.php?latex=%5Ctau_%7Binf%7D#0) | Time required to transfer from state Exposed to state Presymptomatic or Asymptomatic | 4.5 |
| [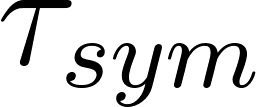](https://latex-staging.easygenerator.com/eqneditor/editor.php?latex=%5Ctau_%7Bsym%7D#0) | Time required to transfer from state Presymptomatic to state Mild | 1.1 |
| [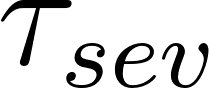](https://latex-staging.easygenerator.com/eqneditor/editor.php?latex=%5Ctau_%7Bsev%7D#0) | Time required to transfer from state Mild to state Severe | 6.6 |
| [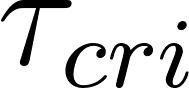](https://latex-staging.easygenerator.com/eqneditor/editor.php?latex=%5Ctau_%7Bcri%7D#0) | Time required to transfer from state Severe to state Critical | 1.5 |
| [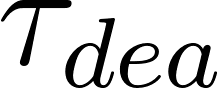](https://latex-staging.easygenerator.com/eqneditor/editor.php?latex=%5Ctau_%7Bdea%7D#0) | Time required to transfer from state Critical to state Dead | 10.7 |
| [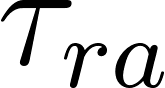](https://latex-staging.easygenerator.com/eqneditor/editor.php?latex=%5Ctau_%7Bra%7D#0) | Time required to transfer from state Asymptomatic to state Recovered | 8 |
| [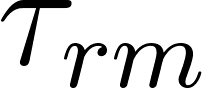](https://latex-staging.easygenerator.com/eqneditor/editor.php?latex=%5Ctau_%7Brm%7D#0) | Time required to transfer from state Mild to state Recovered | 8 |
| [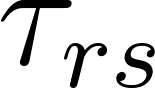](https://latex-staging.easygenerator.com/eqneditor/editor.php?latex=%5Ctau_%7Brs%7D#0) | Time required to transfer from state Severe to state Recovered | 18.1 |
| [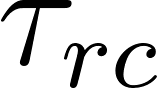](https://latex-staging.easygenerator.com/eqneditor/editor.php?latex=%5Ctau_%7Brc%7D#0) | Time required to transfer from state Critical to state Recovered | 18.1 |

Setting of the Covasim model [[](https://paperpile.com/c/6RLUaH/B0W9U)15[]](https://paperpile.com/c/6RLUaH/B0W9U) was followed.


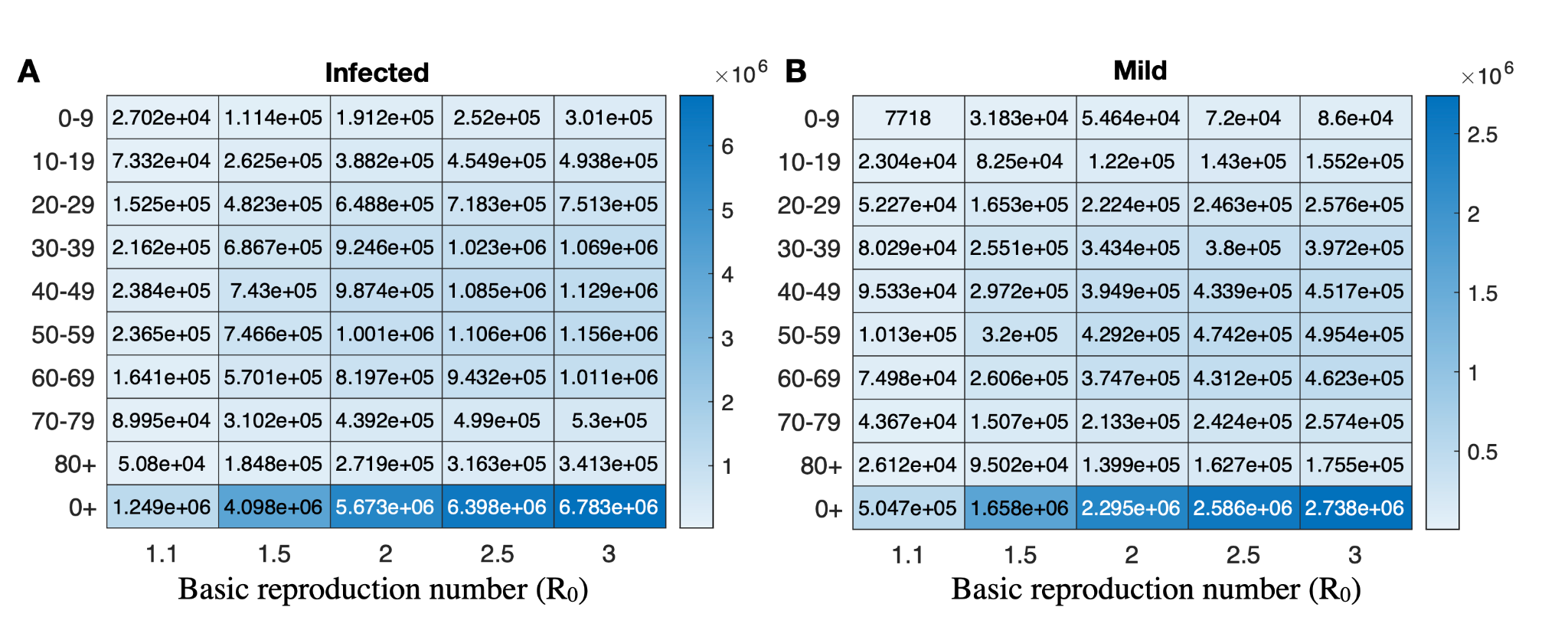


**Figure S1**. Projected age-stratified health burden in Hong Kong. For each basic reproduction number ranging from 1.1 and 3, we estimate the means in (A) total infections and (B) mild infections using COVID-19 epidemic modelling.
